# Prediction of signaling cross-talks contributing to acquired drug resistance in breast cancer cells by Bayesian statistical modeling

**DOI:** 10.1186/s12918-014-0135-x

**Published:** 2015-01-20

**Authors:** AKM Azad, Alfons Lawen, Jonathan M Keith

**Affiliations:** School of Mathematical Science, Monash University, Wellington Road, Clayton, VIC Australia; Department of Biochemistry and Molecular Biology, School of Biomedical Sciences, Monash University, Wellington Road, Clayton, VIC Australia

**Keywords:** Drug resisance, Signaling cross-talk, Bayesian statistical modeling, *p*_1_-model, EGFR signaling, Breast cancer, Lapatinib

## Abstract

**Background:**

Initial success of inhibitors targeting oncogenes is often followed by tumor relapse due to acquired resistance. In addition to mutations in targeted oncogenes, signaling cross-talks among pathways play a vital role in such drug inefficacy. These include activation of compensatory pathways and altered activities of key effectors in other cell survival and growth-associated pathways.

**Results:**

We propose a computational framework using Bayesian modeling to systematically characterize potential cross-talks among breast cancer signaling pathways. We employed a fully Bayesian approach known as the *p*_1_-model to infer posterior probabilities of gene-pairs in networks derived from the gene expression datasets of ErbB2-positive breast cancer cell-lines (parental, lapatinib-sensitive cell-line SKBR3 and the lapatinib-resistant cell-line SKBR3-R, derived from SKBR3). Using this computational framework, we searched for cross-talks between EGFR/ErbB and other signaling pathways from Reactome, KEGG and WikiPathway databases that contribute to lapatinib resistance. We identified 104, 188 and 299 gene-pairs as putative drug-resistant cross-talks, respectively, each comprised of a gene in the EGFR/ErbB signaling pathway and a gene from another signaling pathway, that appear to be interacting in resistant cells but not in parental cells. In 168 of these (distinct) gene-pairs, both of the interacting partners are up-regulated in resistant conditions relative to parental conditions. These gene-pairs are prime candidates for novel cross-talks contributing to lapatinib resistance. They associate EGFR/ErbB signaling with six other signaling pathways: Notch, Wnt, GPCR, hedgehog, insulin receptor/IGF1R and TGF- *β* receptor signaling. We conducted a literature survey to validate these cross-talks, and found evidence supporting a role for many of them in contributing to drug resistance. We also analyzed an independent study of lapatinib resistance in the BT474 breast cancer cell-line and found the same signaling pathways making cross-talks with the EGFR/ErbB signaling pathway as in the primary dataset.

**Conclusions:**

Our results indicate that the activation of compensatory pathways can potentially cause up-regulation of EGFR/ErbB pathway genes (counteracting the inhibiting effect of lapatinib) via signaling cross-talk. Thus, the up-regulated members of these compensatory pathways along with the members of the EGFR/ErbB signaling pathway are interesting as potential targets for designing novel anti-cancer therapeutics.

**Electronic supplementary material:**

The online version of this article (doi:10.1186/s12918-014-0135-x) contains supplementary material, which is available to authorized users.

## Background

Cancer development involves a series of events, ranging from tumorigenesis to metastasis, each of which may be caused by perturbations in crucial signal transduction pathways. Recently, drugs (inhibitors) specifically targeting critical components of signaling pathways known to be up-regulated in specific cancers have been used in the clinic. However, success of these inhibitors is limited by the intrinsic potential of cancer cells to acquire drug resistance. Recent advances in both clinical and laboratory research have reported that cancer cells may adopt several mechanisms against particular treatments including adjusting the signaling circuitry, activation of alternative pathways and cross-talks among various pathways to overcome the effects of inhibitors [1,2]. Resistance to a particular drug such as EGFR (Epidermal Growth Factor Receptor) tyrosine kinase inhibitors, may occur not only due to cross-talks among EGFR-mediated pathways, but also due to cross-talks with pathways triggered by other receptors. Therefore, targeting signaling cross-talks may have the potential to sensitize cancer cells to particular inhibitors.

Drug resistance is a major obstacle in drug efficacy that causes cancer cells to be insensitive to targeted inhibitor therapies and/or conventional chemotherapeutic agents [1,2]. However, there are two categories of resistance to inhibitor therapies: *de novo* and acquired [3]. By definition, *de novo* resistance is a phenotypic characteristic present before drug exposure where drugs with proven efficacy fail to cause tumor cells to respond with any significance [2,4,5]. Acquired resistance refers to a situation where the initial sensitivity of tumor cells to drugs discontinues despite or due to continued consumption [2]. It has been reported that the underlying mechanisms of both types of resistance are related, often due to mutation, loss, or up-regulation of some other important signaling proteins or pathways [2,5]. *De novo* drug resistance can be determined by assessing the genetic profiles of tumors for 1) oncogenic addictions to proteins or pathways and 2) other possible genetic alterations conferring resistance [2]. Therefore, targeting *de novo* resistance can enhance drug efficacy and reduce the chance of acquired resistance [5]. Recently, characterizing drug-resistant tumors, and analyzing cell lines that result from the continuous culture of drug-sensitive cells in the presence of an inhibitor have been shown to be successful approaches for identifying changes responsible for acquired resistance [2].

Cross-talk among signaling pathways may play a vital role in cancer drug resistance, especially in receptor targeted therapies. For example, in EGFR/HER2 signaling pathways, cross-talk with other signaling pathways may occur at various levels of signal transduction: receptor level, mediator level and effector level [1]. At the receptor level, other RTKs (receptor tyrosine kinases) having common downstream targets of EGFR/HER2 may become involved in cross-talk with EGFR/HER2 signaling pathways. In many cancers, these alternative RTKs including MET, IGF1R, FGFR and EphA2 become activated or amplified in order to maintain the signals for cell survival and/or proliferation in common downstream pathways, thus nullifying the inhibition of EGFR kinase [6-10]. Cross-talk at mediator level includes the activation/inactivation of major components of mediator pathways by mutation/deletion of oncogenic driver genes, which eventually activates downstream effectors [1]. These constitutive activations/inactivations of mediator pathways are independent of receptors. The effect of signaling cross-talk in drug resistance at effector level is more complex and diverse since there may be numerous effectors of RTKs signaling pathways. Resistance at the effector level may occur when some critical effectors (i.e. TSC, FOXO3) involved in cell survival and proliferation show an altered phenotype caused by other signaling pathways via RTK signaling cross-talk [1]. Additionally, inhibitor sensitivity can be affected by cross-talk between signaling pathways triggered by the targeted RTK and other signaling pathways (triggered by other RTKs). For example, the EGFR/HER2 signaling pathway can cross-talk with Wnt/ *β*-catenin, Notch, and TNF *α*/IKK/NF- *κ*B signaling pathways to affect the EGFR/HER2 inhibitors’ sensitivities [1]. Cross-talk between effector pathways and feedback inhibition is also responsible for the adaptive and dynamic response of cancer cells against inhibitor therapies, for example, compensating the inhibited components to maintain key downstream functions, such as cell survival, proliferation etc. [11].

Lapatinib is a dual tyrosine kinase inhibitor of EGFR and ErbB2/HER2 receptors [12] that is used in combination therapy of ErbB2/HER2-positive breast cancer patients with advanced or metastatic tumors [13]. Several studies have examined the mechanism underlying lapatinib resistance at the molecular [14-16] and system level [17], active in HER2-positive breast cancer cell-lines through signaling pathways. Garrett *et al.* [14] reported over-expression of *HER2* or *HER3* in lapatinib-resistant SKBR3 and BT474 breast cancer cell lines. Over-expression of AXL tyrosine kinase was found in the BT474 cell-line [16], but interestingly a switched addiction from HER2 to FGFR2 pathway caused the UACC812/LR cell-line to become resistant to lapatinib [15]. Moreover, a detailed analysis of the global cellular network by Komurov *et al.* [17] revealed that up-regulation of the glucose deprivation response pathway compensates for the lapatinib inhibition in SKBR3 cell-line by providing an EGFR/ErbB2-independent mechanism of glucose uptake and survival [17]. Thus, the activation or up-regulation of compensatory pathways confers poor sensitivity of inhibitors (i.e. lapatinib resistance) in EGFR or ErbB2 targeted therapy [1,2,17]. The identification and analyses of potential cross-talks among the signaling pathways may provide deeper insights into the mechanism of drug resistance, and can facilitate finding a range of compensatory pathways for overcoming resistance in targeted therapy.

**Figure 1:**
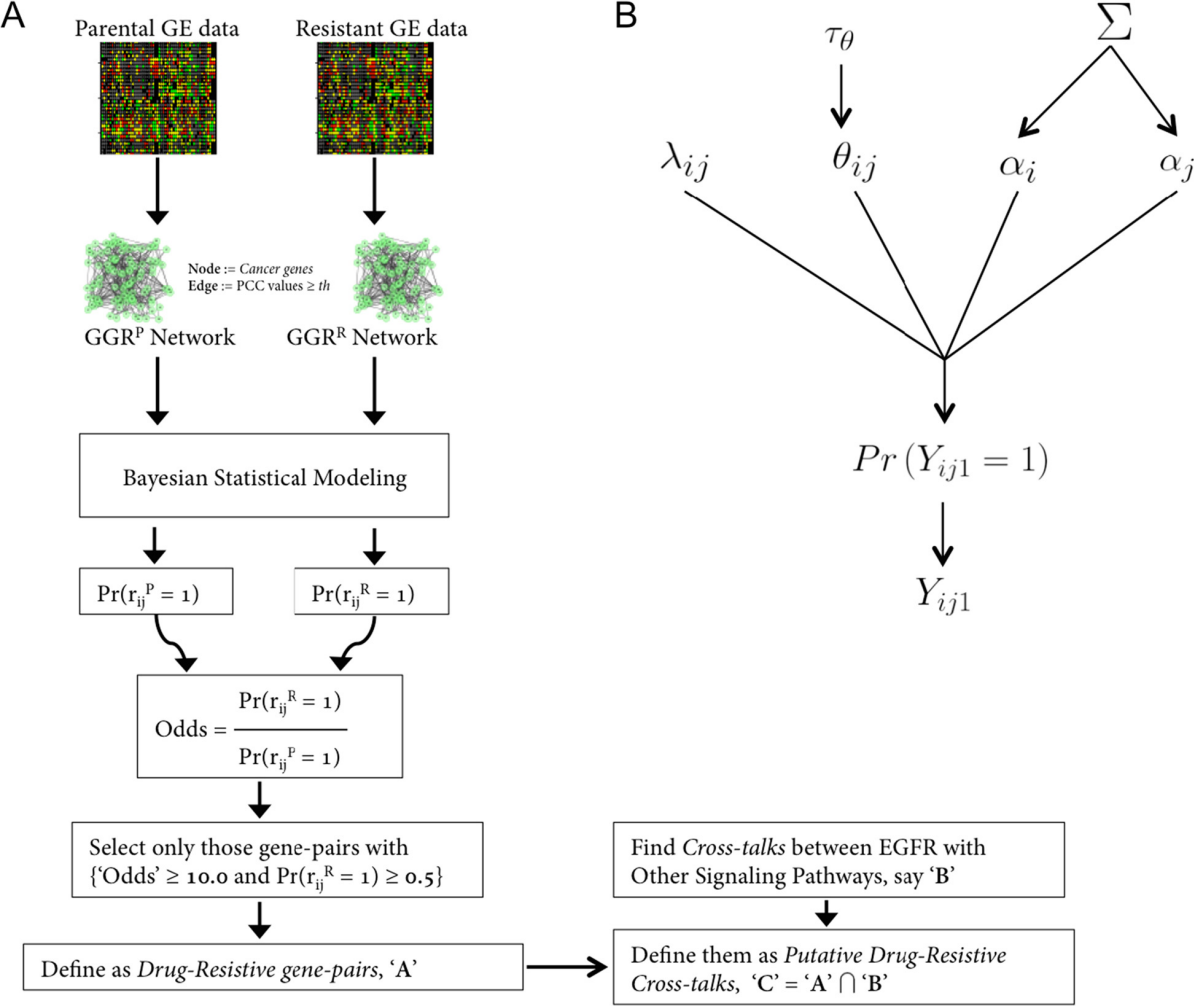
**Schematic diagram of our proposed framework.** **(A)** The framework for finding putative drug-resistant cross-talks. At first two gene expression data matrices were generated individually from the samples of both parental and resistant conditions. Next, based on pair-wise correlations of genes’ expression values, two gene-gene relationship networks were derived. Then, a Bayesian statistical model called the *p*_1_-model was applied on those two networks to find posterior probabilities of network edges. These posterior probabilities were used to find gene-pairs potentially contributing to drug resistance. Next, these gene-pairs were analyzed for overlap with cross-talks between EGFR/ErbB and other signaling pathways, and thus putative drug-resistant cross-talks were identified. **(B)** Hierarchical Bayesian model for inferring posterior probabilities of network parameters. Here, *α* represents the propensity (expansiveness/attractiveness) of a gene to be connected in an undirected network, and is dependent on the hyperparameter *Σ*; *θ* is the global density parameter; *λ*_*ij*_=*l* *o* *g*(*n*_*ij*_) is the scaling parameter, which is fixed due to the constraint $ \sum _{k }^{}{{Y}_{\textit {ijk}}=1} $; the hyperparameter *τ*_*θ*_ represents precision of the normal prior for the parameter *θ*.

In this study, we collected the gene expression values of the ErbB2-positive parental SKBR3 cell-line and the lapatinib-resistant SKBR3-R cell-line, derived from it, in the presence and absence of lapatinib [17]. Then we used a fully Bayesian statistical modeling approach to identify and analyze characteristic drug-resistant cross-talks between EGFR/ErbB and other signaling pathways. ln that process, we considered two gene-gene networks originating from the gene expression matrices of both parental and resistant conditions, individually. To say a gene-pair involved in cross-talk between two particular signaling pathways has high potential of being involved in acquired drug-resistance, our research hypothesis was it should have high probability of appearing in the resistant network and low probability in the parental network. The rationale behind our hypothesis was that in breast cancer cell lines resistant to tamoxifen, a cross-talk mechanism has previously been identified between EGFR and the IGF1R signaling pathway [18]. The schematic diagram of our proposed framework is shown in Figure 1. Like other biological processes, cancer signaling pathway activities and their corresponding network data possess stochasticity such that some gene-gene relationships (i.e. network edges) may not always be present or detected, whereas some other typical relationships may be absent. The stochastic nature of biological systems can be used to predict edge probabilities by formalizing them into a probabilistic model with other network properties [19]. Hill *et al.* reported a data-driven approach that exploits a Dynamic Bayesian Network (DBN) model to infer probabilistic relationships between node-pairs in a context-specific signaling network [20]. This study incorporates existing signaling biology using an informative prior distribution on the network, and its weight of contribution is measured with an empirical Bayes analysis, maximum marginal likelihood. This study predicts a number of known and unexpected signaling links through time that are validated using independent targeted inhibition experiments [20]. Here we have used a fully Bayesian approach for inferring a probabilistic model: a special class of Exponential Random Graph Model, namely the *p*_1_-model. We used Gibbs sampling for estimating model parameters with non-informative priors, in order to estimate the posterior probabilities of edges in gene-gene relationship networks. These identified cross-talks do not appear in the parental network but only in the resistant one, because the signaling network can be ‘rewired’ in a specific context [21,22]. This idea resembles the approach taken by Hill *et al.* in that they inferred the probabilities of signaling links (gene-pairs) varying through time. Thus, these drug-resistance cross-talks can be informative to elucidate the complex mechanisms underlying drug-insensitivity and can help to develop novel therapeutics targeting signaling pathways.

## Materials and method

### Dataset

A global gene expression (GE) dataset (GSE38376) from 1) cells sensitive to lapatinib (said to be under "parental conditions") and 2) cells with acquired resistance to lapatinib was obtained from Komurov *et al.* [17]. Expression values were measured using Illumina HumanHT-12 V3.0 expression beadchip (GPL6947). Samples include SKBR3 parental and resistant (SKBR3-R) each under basal conditions and in response to 0.1 *μ*M and 1 *μ*M lapatinib after 24 hours, where the resistant cell line variant (SKBR3-R) showed 100-fold more resistance to lapatinib treatment than the parental SKBR3 cell line, as reported by Komurov *et al.* [17]. These gene expression datasets used probe-level annotation, which we converted into gene-level annotation. To obtain gene-level GE values, probes were mapped to gene symbols using the corresponding annotation file (GPL6947). While mapping, the average GE values were calculated across all probes if the same gene symbol was annotated to multiple probes. Two GE data matrices were constructed for parental SKBR3 cell lines and resistant SKBR3-R cell lines, respectively, where rows were labelled with gene symbols and columns were labelled with different treatment conditions (0, 0.1 *μ*M and 1 *μ*M of lapatinib).

### Construction of a gene-gene relationship network

We define the gene-gene relationship network as *GGR*:= (*S*,*R*) for each GE data matrix. Here, *S* is a set of 370 cancer related genes collected from the Cancer Gene Census [23]. *R* is defined as the set of pair-wise relationships among seed genes. A gene pair (*gene*_*i*_, *gene*_*j*_) is included in *R* if the corresponding *absolute* Pearson Correlation Coefficient (PCC) is above some threshold, and defined as a pair-wise relationship. These threshold values were empirically chosen for parental and resistant conditions individually, based on the corresponding distributions of all pairwise absolute PCC values. Note PCC values resulting from probes mapped to the same gene were trivially ignored.

### Bayesian statistical modeling of *GGR* network

#### Network model

For statistical modeling of networks, exponential families of distributions offer robust and flexible parametric models [24]. These probabilistic models can be used to evaluate the probability that an edge is present in the network. They can also be used to quantify topological properties of networks by summarizing them in a parametric form and associating sufficient statistics with those parameters [19,24]. In this study, we use a special class of exponential family distributions known as ERGM (Exponential Random Graph Models), also known as the *p*_1_-model, which was introduced by Holland and Leinhardt [24].

A gene-gene relationship network with *g* genes can be regarded as a random variable **X** taking values from a set **G** containing all 2^*g*(*g*−1)^ possible relationship networks [24,25]. Let **u** be a generic point of **G** which can alternatively be denoted as the realization of **X** by **X** = **u**. Let the binary outcome *u*_*ij*_ = 1 if *gene*_*i*_ interacts with *gene*_*j*_, or *u*_*ij*_ = 0 otherwise. Then **u** is a binary data matrix [19]. Let *Pr*(**u**) be the probability function on *G* given by

(1) $$ Pr(u)=Pr(\textbf{X}=\textbf{u})=\frac {1}{\kappa \left(\boldsymbol{\theta}\right)} \exp\sum_{p}^{}{{\boldsymbol{\theta}}_{p}{z}_{p}\left(\textbf{u}\right)}  $$

where *z*_*p*_(**u**) is the network statistic of type *p*, ***θ***_*p*_ is the parameter associated with *z*_*p*_(**u**) and *κ*(***θ***) is the normalizing constant that ensures *Pr*(**u**) is a proper probability distribution (sums to 1 over all **u** in *G*) [26]. The parameter ***θ*** is a vector of model parameters associated with network statistics and needs to be estimated. See [24] for further details.

A major limitation of the *p*_1_-model is the difficulty of calculating the normalizing constant, *κ*(***θ***), since it is a sum over the entire graph space. Estimating the maximum likelihood of this model becomes intractable as there are 2^*g*(*g*−1)^ possible directed graphs (or $2^{\frac {g(g-1)}{2}}$ undirected graphs), each having *g* nodes (genes). A technique called *maximum pseudolikelihood estimation* has been developed to address this problem [27]. This technique employs MCMC methods such as Gibbs or Metropolis-Hastings sampling algorithms [28].

The construction of the *p*_1_-model for a directed network is described in an Appendix Additional file 1: Appendix I. For the gene-gene relationship network with undirected edges, the description of the *p*_1_-model can be simplified by using only two Bernoulli variables *Y*_*ij*0_ and *Y*_*ij*1_ instead of four as follows:

$$ Y_{ijk} = \left\{ \begin{array}{lr} 1 & if \quad u_{ij} = k,\\ 0 & otherwise \end{array} \right. $$

The simplified *p*_1_-model can then be defined using the following two equations to predict the probability of an edge being present between *gene*_*i*_ and *gene*_*j*_:

(2) $$ \log\left\{Pr\left({Y}_{ij1}=1\right) \right\} ={\lambda}_{ij}+\theta +{ \alpha }_{i}+\alpha_{j}  $$

(3) $$ \log\left\{Pr\left({Y}_{ij0}=1\right)\right\} ={\lambda}_{ij}  $$

for *i*<*j*. Note that *λ*_*ij*_ is chosen to ensure *Pr*(*Y*_*ij*0_=1)+*Pr*(*Y*_*ij*1_=1)=1. In this formulation, the expansiveness and attractiveness parameters were reduced to a single parameter, *α*, which represents the propensity of a gene to be connected in an undirected network. Hence, the *p*_1_-model seeks to find the probabilities of edge formation in a network considering its structural features explicitly.

#### Bayesian modeling

We used a fully Bayesian approach for modeling our gene-gene relationship network. Parameter estimation is a crucial step in statistical modeling, for which a classical approach is maximum likelihood estimation (MLE). However, unlike MLE, Bayesian techniques involve calculation of posterior probabilities of model parameters by training the model with given data. We assume that the data  follows the generative model , and assign a prior probability $P\left (\theta |\mathcal {M}\right)$ to the parameter vector *θ* under the model . Then Bayes’ rule for calculating posterior probability is as follows:

(4) $$ Pr\left({\theta}|\mathcal{M},{\mathcal{D}}\right) =\frac {Pr\left(\mathcal{D}|\theta,\mathcal{M}\right) \times Pr\left({\theta}|\mathcal{M}\right)}{\mathcal{Z}}  $$

where $Pr\left (\mathcal {D}|\theta,\mathcal {M}\right)$ is the likelihood function. Now, the marginal likelihood  can be expressed as

(5) $$ \mathcal{Z} = Pr\left(\mathcal{D}|{\mathcal{M}}\right) =\int{Pr\left(\mathcal{D}|{\mathcal{M}},{\theta}\right)\times P\left({\theta}|{\mathcal{M}}\right) d{\theta}},  $$

Computing the exact solution for the marginal likelihood  is often intractable since it is prone to the curse of dimensionality. Fortunately, Markov Chain Monte Carlo (MCMC) methods such as Gibbs sampling and Metropolis-Hastings methods do not require  to be explicitly computed. In general, MCMC methods are stochastic simulation techniques which generate samples from the joint distribution $P\left (\mathcal {M},{\theta }|\mathcal {D}\right)$ for calculating the posterior probabilities of parameters. Here we used Gibbs sampling methods, which sample iteratively, one parameter at a time, from the full conditional distribution given the current and previous values of all other parameters. To implement Gibbs sampling, we employed WinBUGS [29], which is a high-level software package providing an easy interface for implementing complex Bayesian models. In WinBUGS, users are free from background lower-level programming details, and only have to express the model precisely.

We hypothesized that gene-pairs involved in drug resistance are likely to be found with high probabilities in the resistant network but low probabilities in the parental network. Therefore, we built two networks, one from resistant datasets and the other from parental datasets. In this Bayesian approach, the model likelihood is defined in Equations (2) and (3), where *Y*_*k*_ is the data matrix calculated from the observed data **u**. Here we have two *Y*_*k*_ data matrices, namely a gene-gene relationship network *Y*_*k*_^*R*^ derived from resistant samples and *Y*_*k*_^*P*^ derived from parental samples.

Our approach is a hierarchical Bayesian model in that model parameters are in turn dependent on *hyperparameters*. We assign the density parameter *θ* in Equation (2) a normal prior distribution with mean 0 and standard deviation *σ*_*θ*_.

(6) $$ \theta \sim \mathcal{N}\left(0,{{\sigma}_{\theta}}^{2}\right)  $$

Note, in WinBUGS the parameter *τ*, called the *precision*, replaces the standard deviation parameter *σ* of the normal distribution, where, *τ*=*σ*^−2^. For the hyperparameter *τ*_*θ*_ we specify a gamma prior distribution as follows, since it is a conjugate prior for the normal distribution:

(7) $$ {\tau}_{\theta}\sim Gamma\left({a}_{0},b_{0}\right)  $$

We set *a*_0_ = 0.001 and *b*_0_ = 0.001 to make the prior for *θnoninformative*, making its standard deviation wide to express large uncertainty [19]. For attractiveness/ expansiveness parameters *α*_*i*_ and *α*_*j*_, we followed the approach used by Adams *et al.* [30].

(8) $$ \left(\begin{array} {c} {\alpha}_{i}^{R} \\ {\alpha}_{i}^{P} \end{array} \right) \sim \mathcal{N}\left(\left(\begin{array} {c} 0 \\ 0 \end{array} \right),\Sigma \right)  $$

(9) $$ {\Sigma}^{-1}\sim Wishart\left(\left(\begin{array}{cc} 1 & 0 \\ 0 & 1 \end{array} \right),2 \right)  $$

Here, ${\alpha }_{i}^{R} $ and ${\alpha }_{i}^{P}$ represent the expansiveness/attractiveness parameters for the network model of resistant and parental conditions, respectively.

### Drug resistant cross-talk prediction

Since, Lapatinib is an EGFR and ErbB inhibitor, we considered the cross-talks between the EGFR/ErbB signaling pathway and other signaling pathways. Here cross-talks can be defined as any gene-pair (*gene*_*i*_,*gene*_*j*_) in which *gene*_*i*_ ∈ {genes in EGFR/ErbB signaling pathway} and *gene*_*j*_ ∈ {genes in other signaling pathways}, or vice versa [31]. Thus if both genes in any gene-pair were found in the same signaling pathway, that particular gene-pair was trivially ignored. For that purpose, we collected 24 signaling pathways from Reactome [32] (downloaded at 19/05/2014), 35 signaling pathways from KEGG [33,34] (downloaded at 21/10/2014), and 63 signaling pathways from WikiPathway [35] (downloaded at 16/10/2014) databases. Each signaling pathway downloaded from these databases was encoded as tab-delimitated lists of gene symbols.

To determine whether a given gene-pair is involved in drug resistance, we calculated a simple odds ratio of the corresponding two posterior probabilities:

(10) $$ odds=\frac{Pr\left({Y}_{ij1}^{R}=1 \right)}{Pr\left({Y}_{ij1}^{P}=1\right)}  $$

where, ${Y}_{ij1}^{R}$ and ${Y}_{ij1}^{P}$ are gene-gene relationships defined over resistant and parental networks, respectively, and the probabilities are estimated using MCMC sampling. We then selected only those gene-pairs for which the odds score and $Pr\left ({u}_{\textit {ij}}^{R} = 1\right)$ are greater than conservative thresholds, and identified these as the gene-pairs which are potentially involved in drug-resistance.

## Results

### Developing the network

For building gene-gene relationship networks, we considered the genes (nodes) from the Cancer Gene Census [23] only, since our aim was to find those gene-gene relationships which could be potential cross-talks among cancer signaling pathways. In order to identify such gene-pairs, we applied thresholds on their absolute Pearson Correlation Coefficient (PCC) values. These thresholds were 0.545 and 0.54 for parental and resistant conditions, respectively, which we selected from the corresponding distributions of all-pair absolute PCC values with the purpose of considering approximately the top 20% gene-pairs as pairwise relationships only. Applying these thresholds to the relationship values, 27,865 and 26,865 pair-wise relationships were identified in parental and resistant data matrices, respectively.

### Bayesian analysis

For the two gene-gene relationship networks *Y*_*k*_^*R*^ and *Y*_*k*_^*P*^, Bayesian inference of the parameters of the *p*_1_-model for an undirected network was applied. We used WinBUGS for scripting this inference and our scripts were inspired by Adams *et al.* [30]. We used 6000 MCMC iterations for parameter estimation with the first 5000 as ‘burn-in’. All the parameters in the *p*_1_-model appeared to converge rapidly during the burn-in iterations (data not shown). With the above settings, we estimated the posterior probabilities of each edge (gene-gene relationship) *Pr*(*Y*_*ij*1_=1) in the two networks *Y*_*k*_^*R*^ and *Y*_*k*_^*P*^. For each edge, the proportion of the 1000 sampled networks containing the edge was considered as the posterior probability of that edge being present in the network.

Next, for each edge we calculated the odds ratio of their posterior probabilities as defined above. The rationale behind this calculation was that the edges (gene-pairs) found with high probabilities in resistant conditions but lower probabilities in parental conditions are more likely to be due to acquired resistance in cell lines. Therefore, we chose only gene-pairs with high odds ratio (≥ 10.0) and high posterior probabilities (≥ 0.5) of occurring in resistant conditions. We found 11,515 such gene-pairs (Additional file 2: Table S1) among all 68,265 [=(370×369)÷2] possibilities.

**Figure 2:**
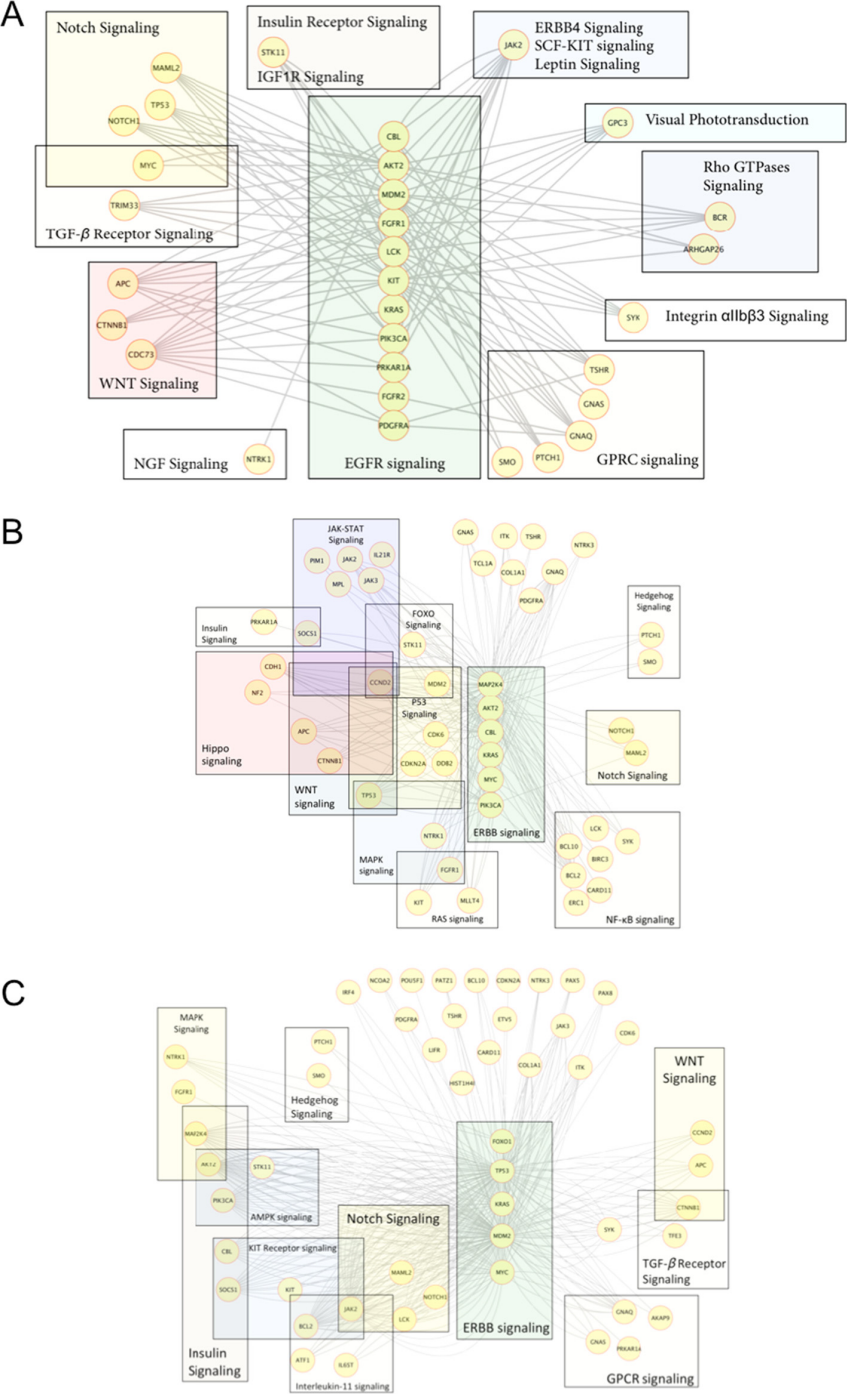
**Network view of (A) 104, (B) 188, and (C) 299 putative drug-resistant cross-talks between pathways using Reactome, KEGG, and WikiPathway pathway databases in Breast Cancer Cell-line: SKBR3 (GSE38376).** Nodes are genes, and the edges are the cross-talks. Note, all the cross-talks here possess posterior probabilities of appearing in resistant network ≥ 0.5 and Odds Ratio ≥ 10.0, which means the posterior probabilities of that cross-talk for appearing in parental network is ≤ 0.05.

We then observed whether the above gene-pairs overlap with the list of potential cross-talks between EGFR/ErbB signaling and other signaling pathways. Here, we collected 24 signaling pathways from Reactome [32], 35 signaling pathways from KEGG [33,34], and 63 signaling pathways from WikiPathway [35] databases, and respectively identified 1,083 (841 distinct), 2,179 (1,050 distinct) and 3,084 (876 distinct) gene-pairs (Additional file 3: Table S2, Additionalfile 4: Table S3 and Additional file 5: Table S4) between EGFR/ErbB and other signaling pathways (seeMaterials and method). Of the 11,515 gene-pairs identified above, we found 104 (97 distinct), 188 (99 distinct) and 299 (96 distinct) gene-pairs overlap with the potential EGFR cross-talks identified using Reactome, KEGG and WikiPathway, respectively. Note the number of potential cross-talks and the number of distinct gene-pairs are different because the same gene-pair can form cross-talks between multiple pathway-pairs (pathways are overlapping). We consider these overlapping gene-pairs as putative drug-resistant cross-talks between EGFR/ErbB and other signaling pathways. In these 104, 188 and 299 cross-talks, we found candidate EGFR/ErbB cross-talks with 13, 26 and 51 other signaling pathways, respectively. Moreover, among all 104, 188 and 299 cross-talks from Reactome, KEGG and WikiPathway, respectively, we found 32 distinct gene-pairs in at least two of these sets. Primary findings and detailed descriptions of all these putative cross-talks from the analyses of all three pathway sources are listed in Table 1, and Additional file 6: Table S5, Additional file 7: Table S6 and Additional file 8: Table S7, respectively. The network views of all these cross-talk sets from the analyses of individual pathway sources are shown in Figure 2.

### Netwalker analyses

**Figure 3:**
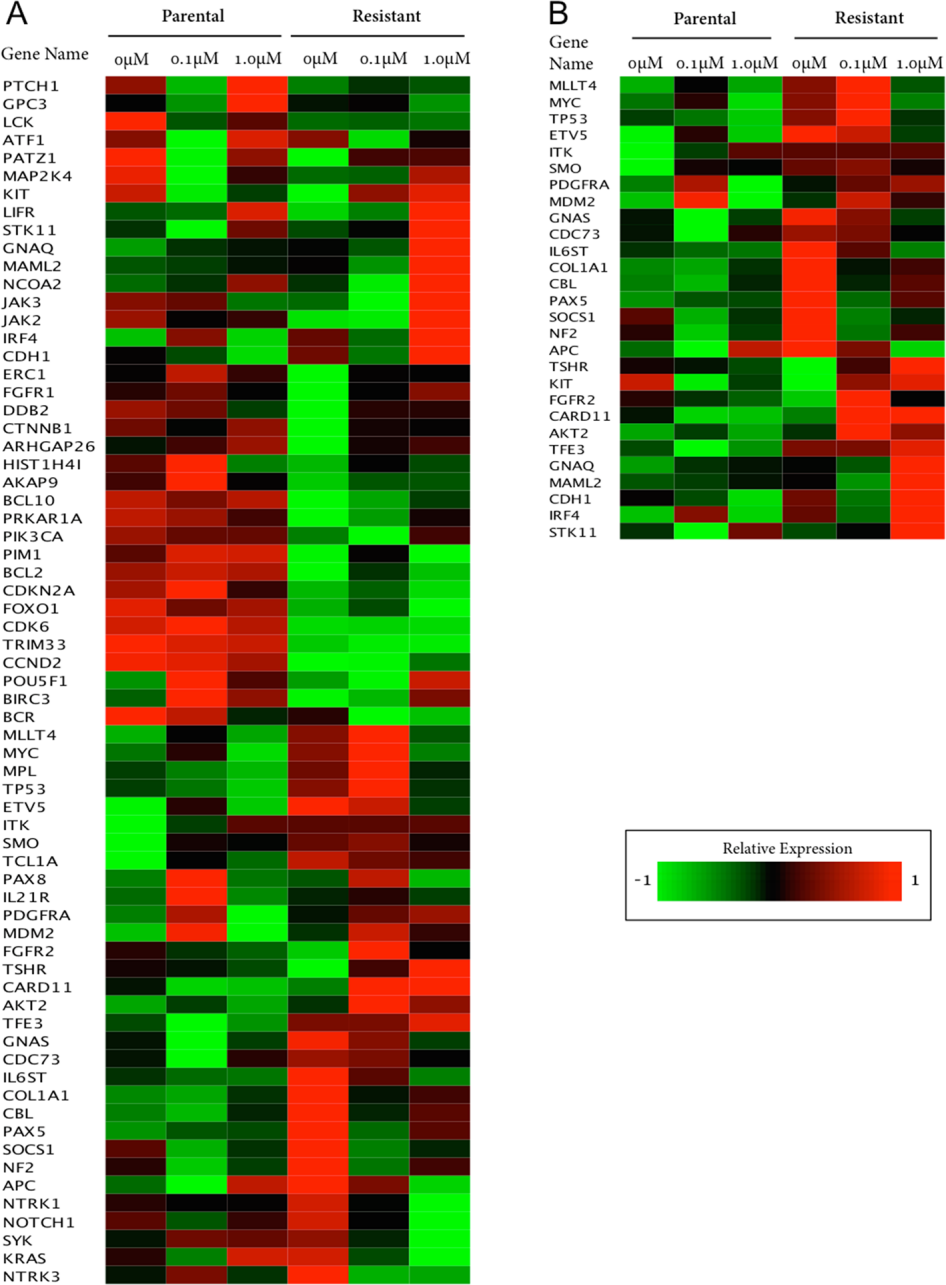
**Heatmap of genes in putative drug-resistant cross-talks in breast cancer cell-line: SKBR3 (GSE38376).** Heatmap image of comparative gene expression changes of parental and resistant conditions in **(A)** all 67 genes in all 104, 188 and 299 putative drug-resistant cross-talks using signaling pathways from Reactome, KEGG and WikiPathway database, respectively, and **(B)** 28 selected genes based on their differential regulation. Here, for each gene, the expression value at each of the 6 conditions (3 parental conditions, and 3 resistant conditions) is the average value of 3 sample patients [17]. For each gene, these 6 expression values (each of them is the average of 3 samples) were transformed into z-scores (zero mean, unit standard deviation) and each z-score was normalized with the maximum absolute value of the z-scores across that particular gene. Note, **(B)** includes only those genes which belonged to gene-pairs for which the average of GE values at resistant conditions was greater than the average of GE values at parental conditions. For both **(A)** and **(B)**, red and green bars indicate up-regulation and down-regulation, respectively.

We conducted further analyses using Netwalker, a network analysis suite for functional genomics [36]. In this analysis, we observed the changes in GE values for each gene in the identified list of potential cross-talks. This was to verify our expectation that, since lapatinib is an EGFR/ErbB inhibitor, both genes involved in drug-resistant cross-talks should be up-regulated in resistant conditions compared to parental conditions, which may imply that the activation of other compensatory signaling pathways in resistant conditions can play a role in acquired resistance to inhibitors by activating the targeted pathway(s) [1,17]. Therefore, for all 67 genes involved in the above sets of 104, 188 and 299 drug-resistant cross-talks from Reactome, KEGG and WikiPathway, respectively, we made a heatmap image of GE values from both conditions (parental and resistant) (Figure 3A). For both resistant and parental conditions, we first averaged the gene expression values from the three samples corresponding to the three treatment conditions. Then these averaged gene expression values were transformed into z-scores (zero mean, unit standard deviation) and each z-score was normalized with the maximum of the absolute values of the z-scores across that particular gene. We observed that in 28 of these 67 genes (involved in 168 cross-talks), gene expression in one or more resistant conditions (0, 0.1 *μ*M and 1 *μ*M of lapatinib) was up-regulated relative to all the parental conditions (0, 0.1 *μ*M and 1 *μ*M of lapatinib) (Figure 3B) which may signify the insensitivity of these genes to inhibitors under resistant conditions. Note for Figure 3B only those genes are depicted for which both genes in some identified cross-talk had average GE values at resistant conditions greater than the average GE values at parental conditions.

**Table 1:** **Primary findings from the analyses using signaling pathways from Reactome, KEGG and WikiPathway in breast cancer cell-line: SKBR3 (GSE38376)**

**Pathway**	**# of signaling**	**Pathway of**	**All**	**Distinct**	**All putative**	**Distinct**	**# of other**
**source**	**pathways**	**interest**	**Cross-talks**	**gene-pairs** ^**§**^	**drug-resistant**	**gene-pairs** ^**¶**^	**signaling**
**of interest**				**cross-talks**		**pathways**	
REACTOME	23	EGFR	1,083	841	104	97	13
KEGG	35	ErbB	2,179	1,050	188	99	26
WikiPathway	63	ErbB	3,084	876	299	96	51

^*¶*^Number of distinct gene-pairs involved in all EGFR/ErbB cross-talks with all other signaling pathways; ^*§*^Number of distinct gene-pairs commonly involved in all EGFR/ErbB cross-talks and drug resistance.

For these 28 selected genes (168 cross-talks), we observed the relative changes in GE values (parental vs resistant conditions) in their candidate signaling pathways. First we analyzed EGFR signaling pathway from Reactome and found that many of the constituent genes were up-regulated in one (or more) resistant conditions whereas in all of their corresponding parental conditions they were down-regulated (Additional file 1: Figure S1). These 168 selected cross-talks associated EGFR (or ErbB) signaling pathways with 6 other signaling pathways that were found in at least two different pathway analyses (i.e. Reactome and KEGG, or KEGG and WikiPathway, or Reactome and WikiPathway). In those 6 other signaling pathways, we also observed a similar phenomenon as above (Additional file 1: Figure S1). These 6 signaling pathways are Notch signaling (in Reactome, KEGG and WikiPathway), Wnt signaling (in Reactome, KEGG and WikiPathway), insulin receptor/IGF1R signaling (in Reactome and WikiPathway), GPCR signaling (in Reactome and WikiPathway), hedgehog (in KEGG and WikiPathway), and TGF- *β* receptor signaling (in Reactome and WikiPathway). Again, for many of the constituent genes of these 6 signaling pathways, expression was up-regulated in at least one of the resistant conditions whereas in all the corresponding parental conditions they were down-regulated. Primary findings regarding these 168 selected drug-resistant cross-talks are listed in Additional file 9: Table S8, and the top 50 of those 168 cross-talks (based on sorted Odds ratio) are shown in Table 2.

**Table 2:** **Description of top 50 (based on sorted Odds ratio) cross-talks among all 168 potential drug-resistant cross-talks between EGFR/ErbB signaling and other pathways from all the analyses using Reactome, KEGG and WikiPathway databases in GSE38376**

**gene**_***i***_ **::gene**_***j***_	**EGFR/ErbB ::**	${Pr\left ({Y}_{ij}^{R }=1 \right)}$	${Pr\left ({ Y }_{ij }^{P }=1 \right)}$	**Odds ratio**	**Avg** $\left ({{GE}_{i}^{P}}\right)$ **:**	**Avg** $\left ({{GE}_{i}^{R}}\right)$ **:**
	**Signaling pathway**_***j***_				**Avg** $\left ({{GE}_{j}^{P}}\right)$	**Avg** $\left ({{GE}_{j}^{R}}\right)$
*AKT2::MAML2* ^§,¶^	Notch signaling	0.5	0.03	16.67	87.71::76.59	96.84::78.6
*MDM2::APC* ^§,$^	Wnt signaling	0.5	0.03	16.67	76.33::82.43	77.9::86.76
*KIT::CDC73* ^§^	Wnt signaling	0.5	0.03	16.67	82.14::104.01	82.68::110.88
*MDM2::CDC73* ^§^	Wnt signaling	0.5	0.03	16.67	76.33::104.01	77.9::110.88
*KIT::GNAQ* ^§^	GPCR signaling	0.5	0.03	16.67	82.14::130	82.68::139.33
*MDM2::GNAQ* ^§,$^	GPCR signaling	0.5	0.03	16.67	76.33::130	77.9::139.33
*KIT::TSHR* ^§^	GPCR signaling	0.5	0.03	16.67	82.14::71.32	82.68::71.66
*MDM2::TSHR* ^§^	GPCR signaling	0.5	0.03	16.67	76.33::71.32	77.9::71.66
*AKT2::APC* ^¶^	Wnt signaling	0.5	0.03	16.67	87.71::82.43	96.84::86.76
*AKT2::APC* ^¶^	Hippo signaling	0.5	0.03	16.67	87.71::82.43	96.84::86.76
*AKT2::CDH1* ^¶^	Hippo signaling	0.5	0.03	16.67	87.71::74.2	96.84::79.8
*AKT2::GNAQ* ^¶^	Gnrh signaling	0.5	0.03	16.67	87.71::130	96.84::139.33
*AKT2::GNAQ* ^¶^	Calcium signaling	0.5	0.03	16.67	87.71::130	96.84::139.33
*AKT2::MDM2* ^¶^	p53 signaling	0.5	0.03	16.67	87.71::76.33	96.84::77.9
*MDM2::AKT2* ^$^	Regulation of toll-like	0.5	0.03	16.67	76.33::87.71	77.9::96.84
	receptor signaling					
*MDM2::AKT2* ^$^	insulin signaling	0.5	0.03	16.67	76.33::87.71	77.9::96.84
*MDM2::AKT2* ^$^	RANKL/RANK signaling	0.5	0.03	16.67	76.33::87.71	77.9::96.84
*MDM2::AKT2* ^$^	AMPK signaling	0.5	0.03	16.67	76.33::87.71	77.9::96.84
*MDM2::AKT2* ^$^	MAPK signaling	0.5	0.03	16.67	76.33::87.71	77.9::96.84
*MDM2::AKT2* ^$^	Tweak signaling	0.5	0.03	16.67	76.33::87.71	77.9::96.84
*MDM2::AKT2* ^$^	Toll-like	0.5	0.03	16.67	76.33::87.71	77.9::96.84
	receptor signaling					
*MDM2::APC* ^$^	BDNF signaling	0.5	0.03	16.67	76.33::82.43	77.9::86.76
*MDM2::APC* ^$^	Wnt signaling Netpath	0.5	0.03	16.67	76.33::82.43	77.9::86.76
*MDM2::APC* ^$^	Wnt signaling	0.5	0.03	16.67	76.33::82.43	77.9::86.76
	and Pluripotency					
*MDM2::COL1A1* ^$^	Nanoparticle-mediated	0.5	0.03	16.67	76.33::91.44	77.9::102.54
	activation of receptor					
	signaling					
*MDM2::COL1A1* ^$^	Osteoblast signaling	0.5	0.03	16.67	76.33::91.44	77.9::102.54
*MDM2::GNAQ* ^$^	TSH signaling	0.5	0.03	16.67	76.33::130	77.9::139.33
*MDM2::GNAQ* ^$^	Serotonin Receptor 2	0.5	0.03	16.67	76.33::130	77.9::139.33
	and STAT3 signaling					
*MDM2::GNAQ* ^$^	Serotonin Receptor 2	0.5	0.03	16.67	76.33::130	77.9::139.33
	and ELK-SRF/GATA4					
	signaling					
*MDM2::ITK* ^$^	T-Cell Receptor and	0.5	0.03	16.67	76.33::89.86	77.9::93.27
	Co-stimulatory signaling					
*MDM2::ITK* ^$^	Tcr signaling	0.5	0.03	16.67	76.33::89.86	77.9::93.27
*MDM2::KIT* ^$^	Kit receptor signaling	0.5	0.03	16.67	76.33::82.14	77.9::82.68
*MDM2::PAX5* ^$^	ID signaling	0.5	0.03	16.67	76.33::68.91	77.9::71.02
*MDM2::TSHR* ^$^	TSH signaling	0.5	0.03	16.67	76.33::71.32	77.9::71.66
*AKT2::TP53* ^§^	Notch signaling	0.5	0.04	12.5	87.71::128.73	96.84::155.09
*KIT::APC* ^§^	Wnt signaling	0.5	0.04	12.5	82.14::82.43	82.68::86.76
*KIT::MAML2* ^§^	Notch signaling	0.5	0.04	12.5	82.14::76.59	82.68::78.6
*KIT::STK11* ^§^	IGF1R signaling	0.5	0.04	12.5	82.14::71.97	82.68::74.95
*KIT::STK11* ^§^	insulin receptor signaling	0.5	0.04	12.5	82.14::71.97	82.68::74.95
*KIT::TP53* ^§^	Notch signaling	0.5	0.04	12.5	82.14::128.73	82.68::155.09
*MDM2::MAML2* ^§,$^	Notch signaling	0.5	0.04	12.5	76.33::76.59	77.9::78.6
*MDM2::STK11* ^§^	IGF1R signaling	0.5	0.04	12.5	76.33::71.97	77.9::74.95
*MDM2::STK11* ^§^	insulin receptor signaling	0.5	0.04	12.5	76.33::71.97	77.9::74.95
*MDM2::TP53* ^§^	Notch signaling	0.5	0.04	12.5	76.33::128.73	77.9::155.09
*AKT2::GNAS* ^¶^	Gnrh signaling	0.5	0.04	12.5	87.71::5465.46	96.84::6212.43
*AKT2::GNAS* ^¶^	Calcium signaling	0.5	0.04	12.5	87.71::5465.46	96.84::6212.43
*AKT2::NF2* ^¶^	Hippo signaling	0.5	0.04	12.5	87.71::85.75	96.84::87.36
*AKT2::TP53* ^¶^	P53 signaling	0.5	0.04	12.5	87.71::128.73	96.84::155.09
*AKT2::TP53* ^¶^	Wnt signaling	0.5	0.04	12.5	87.71::128.73	96.84::155.09
*CBL::CDH1* ^¶^	RAP1 signaling	0.5	0.04	12.5	194.46::74.2	208.45::79.8

Cross-talks found using signaling pathways from ^*§*^Reactome, ^*¶*^KEGG, and ^*$xx*−*xx*^WikiPathway Databases; Pathway _*j*_ is the pathway containing gene _*j*_; $Pr\left ({ Y }_{ij }^{R }=1 \right) $ and $Pr\left ({ Y }_{ij }^{P}=1 \right) $ are the posterior probabilities of gene _*i*_:gene _*j*_ in Resistant and Parental networks, respectively; Avg$\left ({GE}_{i}^{P}\right)$ is the average GE value of all Parental conditions (each of which is an average of 3 samples) for gene _*i*_, Avg$\left ({GE}_{i}^{R}\right)$ is similar but with Resistant conditions, and others are likewise similar.

### Signaling cross-talk between EGFR/ErbB and other signaling pathways

#### Cross-talk between EGFR/ErbB and Notch signaling

We investigated literature evidence regarding the putative cross-talks between EGFR/ErbB signaling and other signaling pathways. We found *AKT2*:*MAML2* (in Reactome and KEGG), *AKT2*:*TP53* (in Reactome), *AKT2*:*MYC* (in Reactome), *KIT*:*MAML2* (in Reactome), *KIT*:*TP53* (in Reactome), *MDM2*:*MAML2* (in Reactome and WikiPathway), *MDM2*:*TP53* (in Reactome), and *TP53*:*MAML2* (in WikiPathway) gene-pairs as putative cross-talks between EGFR/ErbB signaling and Notch signaling pathways. Up-regulation of the Notch signaling pathway inhibits apoptosis and thus contributes to breast carcinogenesis [37]. The Notch signaling pathway cross-talks with EGFR/ErbB signaling at the mediator level [1], e.g. when activated, Notch1 contributes to cell growth and survival via Akt-activation in melanoma [38]. The Notch1 co-activator complex binds to the HES1 promoter [39] which encodes a transcription repressor that represses the expression of PTEN, a PI3K/Akt pathway inhibitor [40] contributing to tyrosine kinase inhibitor (TKI) resistance. Furthermore, Notch1 stimulates *MYC* transcription [41] and this stimulation can lead to the down-regulation of *MYC* via the Akt-pathway [42,43]. This putative gene-pair, *AKT2*:*MYC* was also found in our results as a potential drug-resistant cross-talk between the EGFR/ErbB and TGF- *β* receptor signaling pathways. Again, in HER2/*neu*-mediated resistance to DNA-damaging agents, the Akt pathway becomes activated which eventually suppresses p53 functions via enhancing MDM2-mediated ubiquitination [44]. Protein-protein interaction between MDM2 and p53 is evident as contributing to various cancer related activities [45,46].

#### Cross-talk between EGFR/ErbB and Wnt signaling

We found *MDM2*:*APC* (in Reactome and WikiPathway), *KIT*:*CDC73* (in Reactome), *MDM2*:*CDC73* (in Reactome), *CBL*:*APC* (in Reactome and KEGG), *PDGFRA*:*APC* (in Reactome), and *CBL*:*CDC73* (in Reactome), *AKT2*:*APC* (in KEGG), *AKT2*:*TP53* (in KEGG), and *TP53*:*APC* (in WikiPathway) as putative drug-resistant cross-talks between EGFR/ErbB and Wnt signaling pathways. Deregulation of the Wnt/ *β*-catenin signaling pathway plays a critical role in various cancers including breast, colorectal, pancreatic and colon cancer [47,48], and its association with drug-resistance has been studied by several research groups [47-50]. Recently, it has been reported that resistant cell lines exhibited increased Wnt signaling in both breast and colon cancer [49,50]. Loh *et al.* showed that genes in the Wnt signaling pathway, in both the *β*-catenin dependent (*AXIN2*, *MYC*, *CSNK1A1*) and the independent arms (*ROR2*, *JUN*), were up-regulated in cell lines resistant to tamoxifen compared to the parental MCF7 cell line [49]. Furthermore, *ROR1*, a constituent gene of Wnt signaling pathway, plays a sustainer role in EGFR-mediated prosurvival signaling in lung adenocarcinoma via signaling cross-talk and was therefore reported to be a potential therapeutic target [51]. *APC* and *MDM2* in the *MDM2*:*APC* cross-talk are both tumor suppressors; they co-regulate DNA polymerase- *β* [52,53] which is reported to be hyper-activated in a cis-diamminedichloroplatinum(II) resistant P388 murine leukemia cell line [54]. Again, *β*-catenin whose stability is negatively regulated by *APC* [55], confers resistance to PI3K/Akt inhibitors in colon cancer [56].

#### Cross-talk between EGFR/ErbB and GPCR signaling

Between EGFR/ErbB and GPCR signaling pathways, we found *KIT*:*GNAQ* (in Reactome), *MDM2*:*GNAQ* (in Reactome and WikiPathway), *CBL*:*GNAQ* (in Reactome), *FGFR2*:*GNAQ* (in Reactome), *PDGFRA*:*GNAQ* (in Reactome), *KIT*:*TSHR* (in Reactome), *MDM2*:*TSHR* (in Reactome), *CBL*:*TSHR* (in Reactome), *PDGFRA*:*TSHR* (in Reactome), *KIT*:*GNAS* (in Reactome), *MDM2*:*GNAS* (in Reactome and WikiPathway), *KIT*:*SMO* (in Reactome), *MDM2*:*SMO* (in Reactome), *TP53*:*GNAQ* (in WikiPathway), and *MYC*:*GNAQ* (in WikiPathway). GPCR-like signaling contributes to acquired drug resistance after being mediated by Smoothened (*SMO*) via activating Gli, a canonical hedgehog (Hh) transcription factor [57]. GPCR and EGFR/ErbB over-expression often contributes to cancer growth. Cross-talk between the two at the receptor level contributes to HNSCC (head and neck squamous cell carcinoma) via triggering EGFR/ErbB signaling by a GPCR ligand [58]. For the *MDM2*:*SMO* cross-talk, found between the EGFR/ErbB and GPCR signaling pathways, a *SMO*-mutant from Hh signal transducer activates PI3K/Akt/Gli pathway that eventually increases MDM2 phosphorylation [59]. This in turn increases MDM2-mediated p53 degradation and thus reduces p53-induced apoptosis [59]. Furthermore, recently it has been reported that *SMO* (Hh signal transducer) functions like a G-protein coupled receptor due to its structural resemblance to GPCRs [60,61] which may be further evidence for a drug-resistant cross-talk between hedgehog signaling and EGFR/ErbB signaling [1].

#### Cross-talk between EGFR/ErbB and IR (insulin receptor)/IGF1R signaling

Several studies have reported extensive cross-talk between IR (insulin receptor)/IGF1R (insulin-like growth factor-1 receptor) and EGFR/ErbB signaling pathways contributing to acquired drug resistance in various cancers [62-64]. Loduvini *et al.* reported significant correlation between worse disease-free survival and high co-expression of both EGFR/ErbB and IGF1R in NSCLC (non-small-cell lung cancer) patients [65]. EGFR/ErbB can physically interact with other non-ErbB family receptors at the cell surface and can form heterodimers with receptors like IGF1R, PDGFR etc. [62]. Moreover, the EGFR/ErbB and IGF1R pathways can also cross-talk indirectly via physical interactions between their downstream shared-components [62]. It has been reported recently that gefitinib (an EGFR TKI) inhibits the phosphorylation of IRS1 by IR, but also triggers the association between IRS1 and IGF1R which in turn induces drug-resistance [66]. Knowlden *et al.* showed the cross-talk between IGF1R and EGFR signaling pathways occurred in tamoxifen-resistant MCF7 and T47D breast cancer cell-lines but not in non-resistant cells [18]. Our findings suggest *KIT*:*STK11* (in Reactome), *MDM2*:*STK11* (in Reactome), *MDM2*:*AKT2* (in WikiPathway), *MYC*:*AKT2* (in WikiPathway), *TP53*:*AKT2* (in WikiPathway), *MDM2*:*CBL* (in WikiPathway), *MDM2*:*SOCS1* (in WikiPathway), and *TP53*:*SOCS1* (in WikiPathway) as putative drug-resistant cross-talks between the IGF1R/IR and EGFR/ErbB signaling pathways. For the *MDM2* and *STK11* (also known as *LKB1*) genes, which we identified as a putative cross-talk between EGFR and IGF1R signaling, we did not find any direct supporting evidence in the literature. However, this association is plausible in the resistant conditions given that Yamaguchi *et al.* suggested EGFR signaling may cross-talk with the AMPK/LKB signaling pathway [1]. Moreover, Levine *et al.* reported interconnections between p53 and IGF1R/AKT/mTOR pathways where both *LKB1* and *MDM2* participate in a series of pathway cross-talks [67].

### Validation of the framework using BT474 cell-line (GSE16179)

To further illustrate our method, we analysed a second dataset (GSE16179) containing gene expression profiles of breast cancer cell-line BT474 under two conditions (parental and lapatinib resistant) [16]. The reason for choosing this dataset was that it was obtained using a similar experimental design to the primary dataset GSE38376, but with an additional treatment condition using foretinib (GSK1363089) only and with combined drug use (lapatinib + foretinib). There were three samples per treatment condition. However, to adapt simply and be coherent with the previous experiment, we only considered expression values of parental conditions (3 samples with basal condition: GSM799168, GSM799169 and GSM799170; 3 samples with 1 *μ*M of lapatinib treatment: GSM79917, GSM799172 and GSM799173), and the same conditions with lapatinib resistant cells (3 samples with basal condition: GSM799174, GSM799175 and GSM799176; 3 samples with 1 *μ*M of lapatinib treatment: GSM799180, GSM799181 and GSM799182). Among the 375 cancer genes from Cancer Gene Census [23], there were 357 genes which had gene expression values. We identified 27,358 and 26,292 pair-wise gene-gene relationships (undirected edges) in resistant and parental networks by applying the thresholds 0.71 and 0.81, respectively. Bayesian inference of the *p*_1_-model parameters for an undirected network was applied to these two gene-gene relationship networks as before. Thereafter, among all 63,546 [= (357 ×356) ÷2] possibilities, we found 10,811 gene-pairs (Additional file 10: Table S9) with the same thresholds of odds ratio (≥10.0) as previously, but smaller posterior probability (≥0.15) of occurring in the resistant network. With this set of putative drug-resistant gene-pairs, we also observed the overlap of potential cross-talks of EGFR/ErbB with other signaling pathways using Reactome, KEGG and WikiPathway databases. We found 83 (72 distinct), 133 (87 distinct) and 277 (81 distinct) cross-talks between EGFR/ErbB and other signaling pathways from Reactome, KEGG and WikiPathway (Additional file 11: Table S10, Additional file 12: Table S11 and Additional file 13: Table S12), respectively. The numbers of signaling pathways that were involved in those EGFR/ErbB cross-talks were 10, 18 and 54, respectively. Among the 83, 133 and 277 cross-talks, we found 50 distinct gene-pairs in at least two of these sets. Table 3 shows the comparative findings between our primary dataset (SKBR3 cell-line, GSE38379) and our secondary dataset (BT474 cell-line, GSE16179). In Table 3, we show that some important signaling pathways that were involved in the EGFR/ErbB cross-talks (i.e. Notch, WNT, GPCR, IR/IGF1R, TGF- *β* signaling pathways) in our primary dataset, have some overlap with our secondary dataset.

**Table 3:** **Comparative results between primary dataset (SKBR3 cell-line, GSE38376) and validation dataset (BT474 cell-line, GSE16179)**

**Pathway name**	**Found in Pathway source**	**Found in Pathway source**	**Common cross-talks in both Studies ** ^***¶***^
	**(GSE38376)**	**(GSE16179)**	
Notch Signaling	Reactome,	Reactome,	*MAP2K4::NOTCH1*
	KEGG,	KEGG,	
	WikiPathway	WikiPathway	
GPCR signaling	Reactome,	Reactome,	***CBL::TSHR***
	WikiPathway	WikiPathway	*FGFR1::TSHR*
			***PDGFRA::GNAQ***
			***KIT::TSHR***
			*LCK::TSHR*
			*MDM2::TSHR*
			***PDGFRA::TSHR***
WNT Signaling	Reactome,	Reactome,	*AKT2::CCND2*
	KEGG,	KEGG,	*MAP2K4::CCND2*
	WikiPathway	WikiPathway	*MAP2K4::TP53*
			*MDM2::MAP2K4*
Insulin (IGF1R) Signaling	Reactome,	Reactome,	*MDM2::MAP2K4*
	WikiPathway	WikiPathway	*TP53::MAP2K4*
TGF- *β* Signaling	Reactome,	Reactome,	*MDM2::TFE3*
	WikiPathway	KEGG,	*TP53::TFE3*
		WikiPathway	
MAPK signaling	KEGG,	KEGG,	MDM2::MAP2K4
	WikiPathway	WikiPathway	

^*¶*^These common cross-talks were found using the primary dataset (104, 188 and 299 cross-talks from Reactome, KEGG and WikiPathway databases, respectively) and validation datasets (83, 133 and 277 cross-talks from Reactome, KEGG and WikiPathway databases, respectively). Cross-talks mentioned with **Bold face** are those consistent with our hypothesis that both genes in the particular cross-talk are up-regulated in resistant conditions but down-regulated in parental conditions.

**Figure 4:**
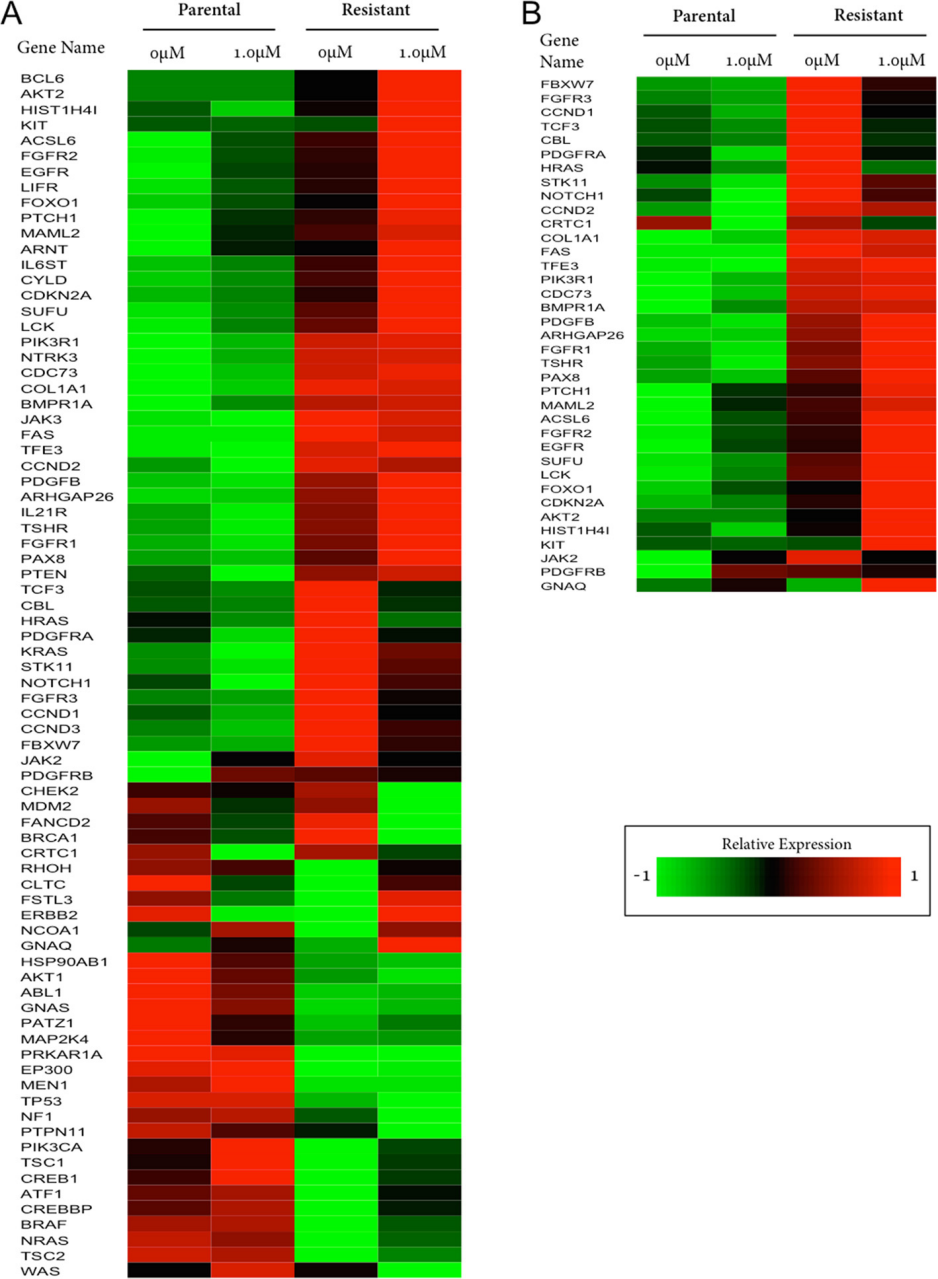
**Heatmap of genes in putative drug-resistant cross-talks in breast cancer cell-line: BT474 (GSE16179).** Heatmap image of comparative gene expression changes of parental and resistant conditions in **(A)** all 78 genes in all 83, 133 and 277 putative drug-resistant cross-talks using signaling pathways from Reactome, KEGG and WikiPathway database, respectively, and **(B)** 37 selected genes based on their differential regulation. Here, for each gene, the expression value at each of the 4 conditions (2 parental conditions, and 2 resistant conditions) is the average value of 3 sample patients [16]. For each gene, these 4 expression values (each of them is the average of 3 samples) were transformed into z-scores (zero mean, unit standard deviation) and each z-score was normalized with the maximum absolute value of the z-scores across that particular gene. For both **(A)** and **(B)**, red and green bars indicate up-regulation and down-regulation, respectively.

There were 78 genes involved in these sets of 83, 133 and 277 putative cross-talks. We performed a similar Netwalker analyses with these 78 genes as we did for the dataset GSE38376, and found 37 genes (involved in 86 cross-talks (Additional file 14: Table S13)) consistent with our hypothesis that both genes in a particular cross-talk should be up-regulated in resistant conditions but down-regulated in parental conditions. In Figure 4, the selected genes from the secondary dataset exhibit an even clearer pattern of up-regulation in resistant conditions than the selected genes from our primary dataset.

## Discussion

In this study, we developed a computational framework to systematically predict signaling cross-talks between EGFR/ErbB and other signaling pathways that contribute to lapatinib (an EGFR and ErbB2/HER2 inhibitor) resistance. We hypothesized that gene-pairs (cross-talks) that can potentially cause drug-resistance have a high probability of occurring in the resistant condition(s) but a low probability in parental conditions. We employed a fully Bayesian statistical model: a special class of Exponential Random Graph Model known as the *p*_1_-model, to infer the posterior probabilities of such gene-pairs from corresponding networks inferred using gene expression values [17] of resistant and parental conditions. In selecting gene-pairs as putative cross-talks, threshold values for two parameters: odds and posterior probabilities of edges in resistant networks were empirically selected. However, more robust procedures for the selection of these two parameters can be made in future studies. All other parameters in the *p*_1_-model discussed above were estimated using Gibbs sampling (see Materials and method).

Our results primarily focus on compensatory signaling pathways i.e. Notch signaling, Wnt signaling, GPCR signaling, and IR/IGF1R signaling, which cross-talk with EGFR/ErbB signaling to reduce the inhibiting effect of lapatinib. We present additional literature evidence that the identified cross-talks of the above compensatory signaling pathways with EGFR/ErbB signaling may contribute to drug-resistance by maintaining key cell survival and/or proliferation signals in common down-stream pathways, including PI3K/Akt signaling [1].

Komurov *et al.* [17] hypothesized that cross-talks between EGFR/ErbB signaling and metabolic pathways contribute to resistance to lapatinib. More specifically, they identified that glucose deprivation reduces the inhibiting effects of lapatinib by up-regulating constituent genes and thus providing an EGFR/ErbB2-independent mechanism of glucose uptake and cell survival [17]. Here, by using the same gene expression datasets, we found *MDM2*:*STK11* cross-talk between EGFR/ErbB and IGF1R signaling, where STK11 (also known as *LKB1*) phosphorylates and activates *AMPK* in absence of glucose [67]. Again, in the integrated signaling circuitry of pathways: p53-IGF-1-AKT-TSC2-mTOR, a positive feedback loop (p53-PTEN AKT-MDM2-p53) is formed which enhances p53-mediated apoptosis and senses nutrient deprivation [67]. Thus our results complement the findings of Komurov *et al.* by finding *signaling* cross-talks between EGFR/ErbB and IGF1R pathways.

In Netwalker analysis of our primary dataset (SKBR3 cell-line, GSE38376), we compared the expression changes of all the samples in parental conditions (basal, 0.1 *μ*M and 1.0 *μ*M) with those of all the samples in resistant conditions (basal, 0.1 *μ*M and 1.0 *μ*M). However, we conducted another experiment on both of our primary (SKBR3 cell-line, GSE38376) and secondary datasets (BT474 cell-line, GSE16179) in which we first identified genes dysregulated in treatment vs basal conditions in parental samples and then checked if those genes were reversely changed in treatment conditions in resistant samples. To that end, for each sample, first we calculated the fold-change(s) of parental treatment condition(s) compared to parental basal condition, and then we calculated the fold-changes of resistant basal and resistant treatment conditions compared to parental basal condition (Additional file 1: Figure S2A and S3A). Then, we chose only those genes for which, in any of the 3 samples, expressions were dysregulated (up-/down-regulated) in (all the) parental treatment condition(s) (*log*_2_ of fold-changes were positive/negative), and for that particular sample, expressions were reversely changed (the fold-change sign was opposite to that of parental condition) in all the resistant treatment conditions (Additional file 1: Figure S2B and S3B). This may be a strong indicator of sensitivity to an inhibitor in parental conditions and the development of acquired resistance. Next, we compared these selected genes to cross-talks found in results from GSE38379 (104, 188 and 299 EGFR/ErbB cross-talks from Reactome, KEGG and WikiPathway, respectively) and GSE16179 (83, 133 and 277 EGFR/ErbB cross-talks from Reactome, KEGG and WikiPathway, respectively). Although we didn’t find any such cross-talks overlapping with the results from the primary dataset (GSE38379), we found 401 from our secondary dataset (GSE16179) (Additional file 15: Table S14).

Currently, our network modeling only considers undirected edges among genes. In future we would like to generalise the approach to identify directed and indirect interactions among genes. In network modeling, a combination of both direct and indirect relationships among gene-pairs was found to provide better insights into biological systems in our previous studies [68]. The rationale for combining these two types of gene-gene relationships in signaling networks is that EGFR/ErbB and IGF1R can both cross-talk (EGFR/IGF1R heterodimerization) directly at the receptor level, and indirectly mediated by GPCR signaling, as reported by Van der Veeken *et al.* [62]. Other high-throughput datasets such as miRNA expression data, copy number aberration data, and methylation data could also be incorporated into our framework to obtain a better understanding of gene dependencies. Note that our methodology exploits a fully data-driven approach for finding putative drug-resistant cross-talks, without incorporating other prior information regarding gene-gene relationships, such as Protein-Protein Interactions. Hence, although our data-driven approach may inherently yield some false-positive predictions, it may also provide the possibilities of finding novel cross-talks contributing to drug- resistance.

## Conclusions

Our proposed computational framework is able to predict putative cross-talks among signaling pathways that play a role in drug resistance in two breast cancer cell-lines, SKBR3 and BT474. Our framework could also be useful for other types of cancer to enhance understanding of the role of signaling cross-talks in drug resistance. Most importantly, we believe our method can be used to find a range of compensatory pathways that nullify/reduce the inhibiting effects of drugs via cross-talk with targeted pathways. These novel compensatory pathways can be further considered as novel targets for single or combination therapies.

## Additional files

Additional file 1
**Appendix I.** Derivation of *p*_1_-model for directed network.

Additional file 2
**Table S1.** All 11,515 drug-resistant gene-pairs found in GSE38376.

Additional file 3
**Table S2.** All 1,083 (841 distinct) cross-talks found between EGFR and other 23 signaling pathways from Reactome database.

Additional file 4
**Table S3.** All 2,179 (1,050 distinct) cross-talks found between ErbB and other 34 signaling pathways from KEGG database.

Additional file 5
**Table S4.** All 3,084 (876 distinct) cross-talks found between ErbB and other 62 signaling pathways from WikiPathway database.

Additional file 6
**Table S5.** 104 drug-resistant cross-talks found between EGFR and other 23 signaling pathways from Reactome database [GSE38376].

Additional file 7
**Table S6.** 188 drug-resistant cross-talks found between ErbB and other 34 signaling pathways from KEGG database [GSE38376].

Additional file 8
**Table S7.** 299 drug-resistant cross-talks found between ErbB and other 62 signaling pathways from WikiPathway database [GSE38376].

Additional file 9
**Table S8.** 168 selected cross-talks which associated EGFR (or ErbB) signaling pathways with 6 other signaling pathways that were found in at least two different pathway analyses (i.e. Reactome and KEGG, or KEGG and WikiPathway, or Reactome and WikiPathway) [GSE38376].

Additional file 10
**Table S9.** All 10,811 drug-resistant gene-pairs found in GSE16179.

Additional file 11
**Table S10.** 83 drug-resistant cross-talks found between EGFR and other 23 signaling pathways from Reactome database [GSE16179].

Additional file 12
**Table S11.** 133 drug-resistant cross-talks found between ErbB and other 34 signaling pathways from KEGG database [GSE16179].

Additional file 13
**Table S12.** 278 drug-resistant cross-talks found between ErbB and other 62 signaling pathways from WikiPathway database [GSE16179].

Additional file 14
**Table S13.** 86 drug-resistant cross-talks found in all Reactome, KEGG and WikiPathway analyses where both genes in a particular cross-talk was up-regulated in resistant conditions but down-regulated in parental conditions [GSE16179].

Additional file 15
**Table S14.** 401 cross-talks from Reactome, KEGG and WikiPathway analyses where the genes are dysregulated in parental treatment vs parental basal condition, and reversely changed in resistant basal + resistant treatment vs parental basal condition [GSE16179].
