# Supplementary material for: Prediction of signaling cross-talks contributing to acquired drug resistance in breast cancer cells by Bayesian statistical modeling
Source: BMC Syst Biol. 2015 Jan 20;9:2. doi: 10.1186/s12918-014-0135-x (PMC4307189; doi:10.1186/s12918-014-0135-x)
Supplement: Additional file 1 — Appendix I. Derivation of p 1-model for directed network. [file 12918_2014_135_MOESM1_ESM.pdf]

Additional File 1: Prediction of Signaling Cross-talks Contributing to Acquired Drug Resistance in Breast Cancer Cells by Bayesian Statistical Modeling

A. K. M. Azad[1,*], Alfons Lawen[2], Jonathan.Keith[1]
[1]School of Mathematical Science, Monash University
[2]Department of Biochemistry and Molecular Biology, School of Biomedical Sciences, Monash University
*E-mail: aaza7@student.monash.edu

Appendix I: Derivation of p_1-model for directed network

Let $\mathbf{X}$ be a directed network with n nodes and a realization of of that network is represented as $\mathbf{X} = \mathbf{u}$. Let the binary outcome $u_{ij} = 1$ if $gene_i$ interacts with $gene_j$, or $u_{ij} = 0$ otherwise. Then $\mathbf{u}$ is a binary data matrix [1]. Let $Pr(\mathbf{u})$ be the probability function on G given by

$$Pr(u) = Pr(\mathbf{X} = \mathbf{u}) = \frac{1}{\kappa(\boldsymbol{\theta})} exp \sum_p \boldsymbol{\theta}_p z_p(\mathbf{u}) \tag{1}$$

where $z_p(\mathbf{u})$ is the network statistic of type p, $\boldsymbol{\theta}_p$ is the parameter associated with $z_p(\mathbf{u})$ and $\kappa(\boldsymbol{\theta})$ is the normalizing constant that ensures $Pr(\mathbf{u})$ is a proper probability distribution (sums to 1 over all $\mathbf{u}$ in G) [2]. The parameter $\boldsymbol{\theta}$ is a vector of model parameters associated with network statistics and needs to be estimated. See [3] for further details.

Derivation: The p_1-model considers the joint distribution of dyads $D_{ij} = (u_{ij}, u_{ji})$ with dyadic probabilities

$$m_{ij} = Pr(mutual \quad dyad) = Pr\{D_{ij} = (1,1)\}; \quad \forall (i < j), \tag{2}$$

$$a_{ij} = Pr(asymmetric \quad dyad) = Pr\{D_{ij} = (1,0)\}; \quad \forall (i \neq j), \tag{3}$$

$$n_{ij} = Pr(null \quad dyad) = Pr\{D_{ij} = (0,0)\}; \quad \forall (i < j), \tag{4}$$

and

$$m_{ij} + a_{ij} + a_{ji} + n_{ij} = 1; \quad \forall (i < j). \tag{5}$$

This model finds the probabilities of each type of dyadic relation for each pair of genes. Assuming all the dyads D_{ij} are statistically independent, the probability distribution of $\mathbf{X} = \mathbf{u}$ can be specified as the joint distribution of the dyads (such as, D_{12}, D_{13}, and so on) which may be expressed in the following way:

$$Pr(\boldsymbol{X} = \boldsymbol{u}) = \prod_{i<j} m_{ij}^{u_{ij}u_{ji}} \prod_{i \neq j} a_{ij}^{u_{ij}(1-u_{ji})} \prod_{i<j} n_{ij}^{(1-u_{ij})(1-u_{ji})} \tag{6}$$

In order to get an exponential form, the above equation can be reexpressed as follows:

$$Pr(\boldsymbol{X} = \boldsymbol{u}) = exp\left\{\sum_{i<j} \rho_{ij} u_{ij} u_{ji} + \sum_{i \neq j} \theta_{ij} u_{ij}\right\} \prod_{i<j} n_{ij}, \tag{7}$$

where

$$\rho_{ij} = \log\left(\frac{m_{ij} n_{ij}}{a_{ij} a_{ji}}\right); \quad \forall (i < j) \tag{8}$$

and

$$\theta_{ij} = \log\left(\frac{a_{ij}}{n_{ij}}\right); \quad \forall\left(i \neq j\right) \tag{9}$$

Note that we interpret $n_{ij} = n_{ji}$ for i > j. The parameter ρ_{ij} is a log-odds ratio which measures the force of reciprocation of the edge between $gene_i$ and $gene_j$. By doing simple algebra as follows, it can be said that ρ_{ij} specifies the log of increase in the odds that $u_{ij} = 1$ given $u_{ji} = 1$

$$exp\left(\rho_{ij}\right) = \left\{\frac{Pr\left(u_{ij} = 1|u_{ji} = 1\right)}{Pr\left(u_{ij} = 0|u_{ji} = 1\right)}\right\} / \left\{\frac{Pr\left(u_{ij} = 1|u_{ji} = 0\right)}{Pr\left(u_{ij} = 0|u_{ji} = 0\right)}\right\} \tag{10}$$

The parameter θ_{ij}, is also a log-odds ratio which measures the probability of an asymmetric edge between $gene_i$ and $gene_j$ with $u_{ji} = 0$. This intuition can be explained by the following calculation:

$$exp\left(\theta_{ij}\right) = \frac{Pr\left(u_{ij} = 1|u_{ji} = 0\right)}{Pr\left(u_{ji} = 0|u_{ij} = 0\right)} \tag{11}$$

Equation (7) provides a more general family of distributions for **X** than Equation (1). However, Equation (7) contains too many parameters; therefore, restrictions were applied on the parameters, ρ_{ij} and θ_{ij} to obtain Equation (1) from Equation (7). Thus, the original p_1-model was postulated as following:

$$\rho_{ij} = \rho; \quad \forall\left(i < j\right), \tag{12}$$

and

$$\theta_{ij} = \theta + \alpha_i + \beta_j; \quad \forall\left(i \neq j\right), \tag{13}$$

Here, ρ indicates the global degree of reciprocity of the entire network; θ is the global density parameter; α_i is a local parameter measuring the *expansiveness* of $gene_i$ which is the propensity of $gene_i$ to send edges; and β_j represents the *attractiveness* of $gene_j$ which is the ability of $gene_j$ to attract edges. Based on the transformation of parameters in Equations (12) and (13), the distribution formula for the p_1-model can be rewritten as follows:

$$Pr\left(\boldsymbol{X} = \boldsymbol{u}\right) = exp\left\{\sum_{i<j}\rho M + \theta E + \sum_{i}\alpha_i \triangle_{out}\left(i\right) + \sum_{j}\beta_j \triangle_{in}\left(j\right)\right\}\prod_{i<j} n_{ij} \tag{14}$$

The above form of the p_1-model equation represents the exponential family of distributions with the following statistics: M - the number of reciprocated edges, E - total number of edges, and $\triangle_{out}\left(i\right)$ and $\triangle_{in}\left(i\right)$ - the in- and out-degree of $gene_i$. To facilitate Gibbs sampling, an equivalent log-linear formulation of the p_1-model was suggested by Fienberg and Wasserman [4]. In this formulation, a dyad (u_{ij},u_{ji}) is represented by four Bernoulli variables Y_{ij00}, Y_{ij10}, Y_{ij01} and Y_{ij11} as follows:

$$Y_{ijkl} = \begin{cases} 1 & if \quad u_{ij} = k, u_{ji} = l, \\ 0 & \qquad otherwise \end{cases}$$

Then, the p_1-model can be expressed with four log-linear equations:

$$log\left\{Pr\left(Y_{ij10} = 1\right)\right\} = \lambda_{ij} + \theta + \alpha_i + \beta_j \tag{15}$$

$$log\left\{Pr\left(Y_{ij01} = 1\right)\right\} = \lambda_{ij} + \theta + \alpha_j + \beta_i \tag{16}$$

$$log\left\{Pr\left(Y_{ij11} = 1\right)\right\} = \lambda_{ij} + 2\theta + \alpha_i + \alpha_j + \beta_i + \beta_j + \rho \tag{17}$$

$$log\left\{Pr\left(Y_{ij00} = 1\right)\right\} = \lambda_{ij} \tag{18}$$

for $i < j$. Here, $\lambda_{ij} = log\left(n_{ij}\right)$ is the scaling parameter, which is fixed due to the constraint $\sum_{k,l} Y_{ijkl} = 1$.

References

1. Bulashevska, S., Bulashevska, A., Eils, R.: Bayesian statistical modelling of human protein interaction network incorporating protein disorder information. BMC Bioinformatics **11**, 46 (2010)

2. Wasserman, S., Pattison, P.: Logit models and logistic regressions for social networks: I. an introduction to markov graphs andp. Psychometrika **61**(3), 401–425 (1996)

3. Holland, P.W., Leinhardt, S.: An exponential family of probability distributions for directed graphs. Journal of the American Statistical Association **76**(373), 33–50 (1981)

4. Fienberg, S., Wasserman, S.: Categorical data analysis of single sociometric relations. Sociological Methodology **12**, 156–192 (1981)

1 Supplementary Figures

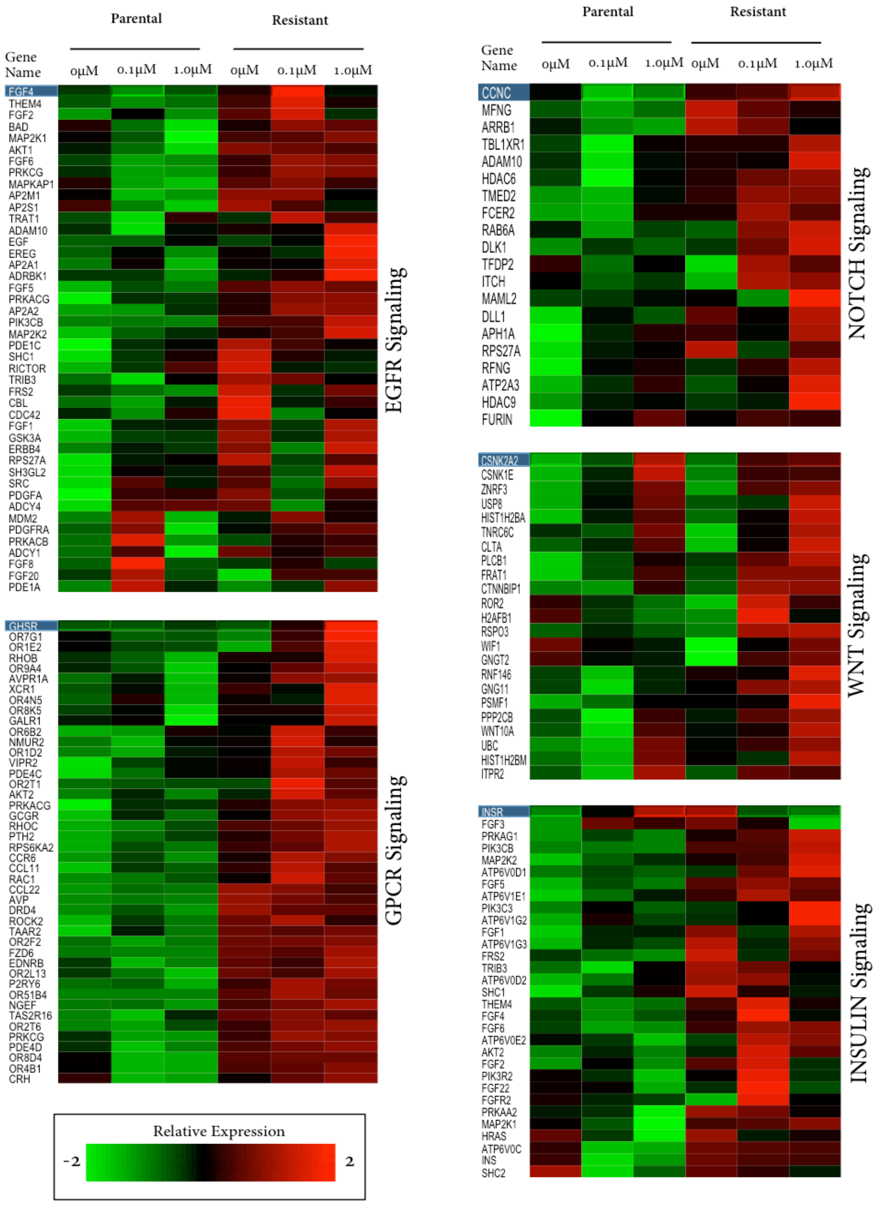

Figure 1. Comparative expression changes in parental and resistant conditions (SKBR3 cell-line, GSE38376) of some constituent genes of EGFR, GPCR, Notch, Wnt and insulin signaling. Expression of these genes in parental conditions is down-regulated but up-regulated in resistant conditions which signify the effect of drug resistance on those genes. Here, pathway annotations are from Reactome database.

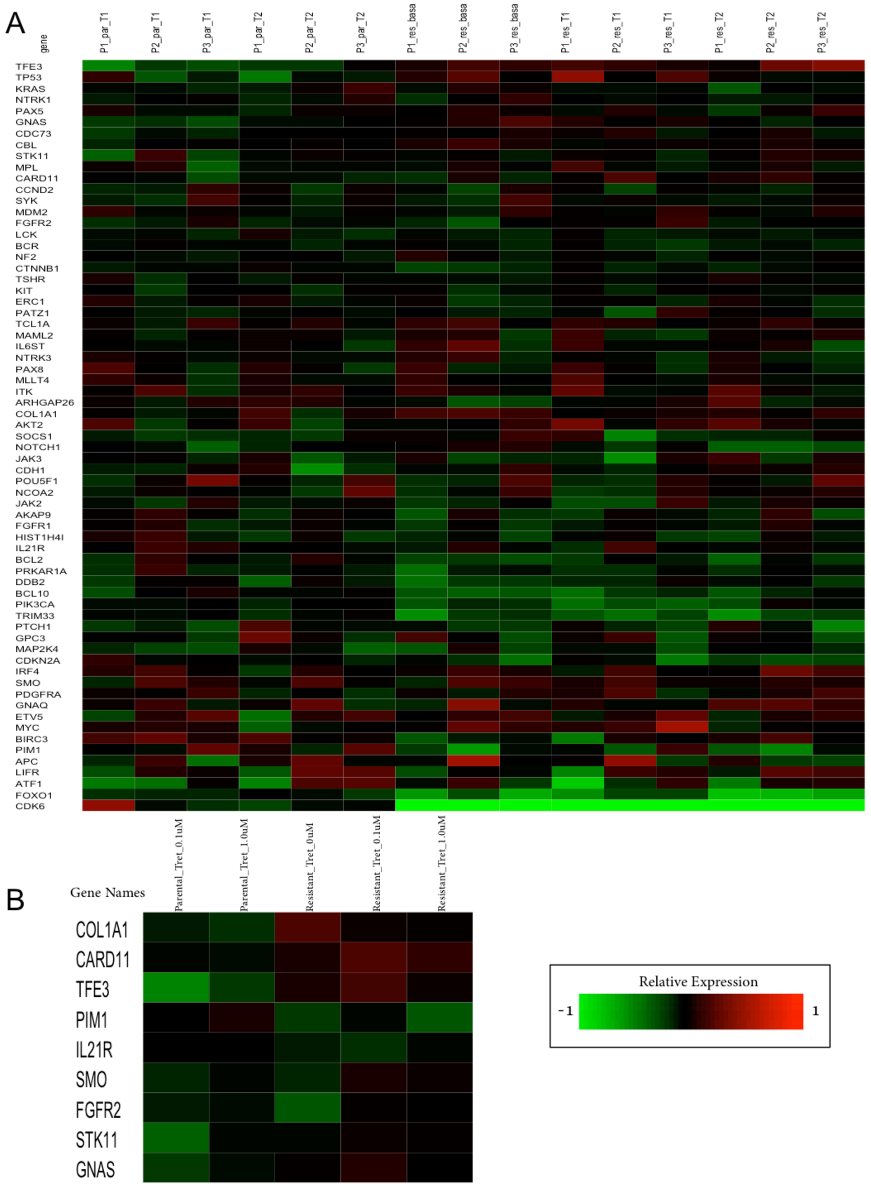

Figure 2. Fold change analysis of gene expressions in both parental and resistant conditions compared to parental basal condition (0 μM) in our primary dataset (SKBR3 cell-line, GSE38376). (A) Genes depicted here are from 104, 188 and 299 EGFR/ErbB cross-talks found using signaling pathways from Reactome, KEGG and WikiPathway databases, respectively **(B)** List of those genes that are dysregulated in parental treatment vs parental basal condition and reversely changed in resistant basal + resistant treatment vs parental basal condition. Each column here represents each condition's gene expression fold-change of either, one of the parental treatment conditions (0.1 μM, 1.0 μM), or one of the resistant conditions (0 μM, 0.1 μM, 1.0 μM) compared to parental basal treatment condition (0 μM).

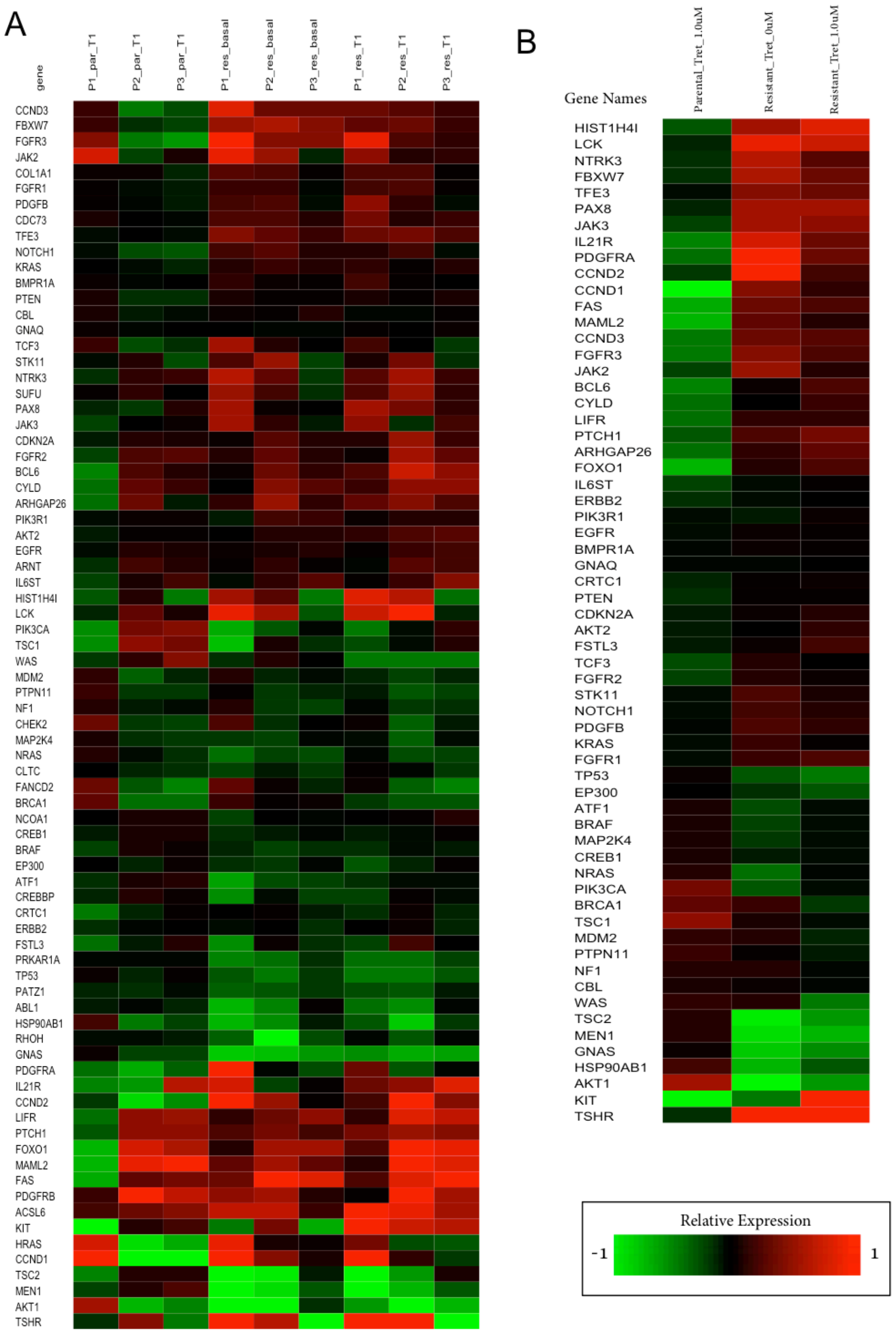

Figure 3. Fold change analysis of gene expressions in both parental and resistant conditions compared to parental basal condition (0 μM) in our validation dataset (BT474 cell-line, GSE16179). (A) Genes depicted here are from 83, 133 and 278 EGFR/ErbB cross-talks found using signaling pathways from Reactome, KEGG and WikiPathway databases, respectively **(B)** List of those genes that are dysregulated in parental treatment vs parental basal condition and reversely changed in resistant basal + resistant treatment vs parental basal condition. Each column here represents each condition's gene expression fold-change of either, one of the parental treatment conditions (0.1 μM, 1.0 μM), or one of the resistant conditions (0 μM, 0.1 μM, 1.0 μM) compared to parental basal treatment condition (0 μM).
